# Intravitreal autologous mesenchymal stem cell transplantation: a non-randomized phase I clinical trial in patients with retinitis pigmentosa

**DOI:** 10.1186/s13287-020-02122-7

**Published:** 2021-01-09

**Authors:** Aekkachai Tuekprakhon, Siripakorn Sangkitporn, Adisak Trinavarat, Aulia Rahmi Pawestri, Visit Vamvanij, Monchai Ruangchainikom, Panya Luksanapruksa, Phitchapa Pongpaksupasin, Areerat Khorchai, Acharaporn Dambua, Patcharaporn Boonchu, Chonlada Yodtup, Mongkol Uiprasertkul, Somchai Sangkitporn, La-ongsri Atchaneeyasakul

**Affiliations:** 1 Department of Ophthalmology, Faculty of Medicine Siriraj Hospital, Mahidol University, 2 Wanglang Road, Bangkok Noi, Bangkok, 10700 Thailand; 2 Stem cell and Regenerative Medicine Center, Department of Medical Sciences, Ministry of Public Health, National Institute of Health, 88/7 Tivanon Road, Muang, Nonthaburi, 11000 Thailand; 3 Faculty of Medicine, Universitas Brawijaya, Malang, Indonesia; 4 Department of Orthopaedic Surgery, Faculty of Medicine Siriraj Hospital, Mahidol University, Bangkok, Thailand; 5 Research Department, Faculty of Medicine Siriraj Hospital, Mahidol University, Bangkok, Thailand; 6 Department of Pathology, Faculty of Medicine Siriraj Hospital, Mahidol University, Bangkok, Thailand

**Keywords:** Retinitis pigmentosa (RP), Inherited retinal diseases, Mesenchymal stem cell, Stem cell therapy, Phase I clinical trial

## Abstract

**Background:**

Retinitis pigmentosa (RP) is a progressive inherited retinal disease with great interest for finding effective treatment modalities. Stem cell-based therapy is one of the promising candidates. We aimed to investigate the safety, feasibility, and short-term efficacy of intravitreal injection of bone marrow-derived mesenchymal stem cells (BM-MSCs) in participants with advanced stage RP.

**Methods:**

This non-randomized phase I clinical trial enrolled 14 participants, categorized into three groups based on a single dose intravitreal BM-MSC injection of 1 × 10^6^, 5 × 10^6^, or 1 × 10^7^ cells. We evaluated signs of inflammation and other adverse events (AEs). We also assessed the best corrected visual acuity (BCVA), visual field (VF), central subfield thickness (CST), and subjective experiences.

**Results:**

During the 12-month period, we noticed several mild and transient AEs. Interestingly, we found statistically significant improvements in the BCVA compared to baseline, although they returned to the baseline at 12 months. The VF and CST were stable, indicating no remarkable disease progression. We followed 12 participants beyond the study period, ranging from 1.5 to 7 years, and observed one severe but manageable AE at year 3.

**Conclusion:**

Intravitreal injection of BM-MSCs appears to be safe and potentially effective. All adverse events during the 12-month period required observation without any intervention. For the long-term follow-up, only one participant needed surgical treatment for a serious adverse event and the vision was restored. An enrollment of larger number of participants with less advanced RP and long-term follow-up is required to evaluate the safety and efficacy of this intervention.

**Trial registration:**

ClinicalTrials.gov, NCT01531348. Registered on February 10, 2012

**Supplementary Information:**

The online version contains supplementary material available at 10.1186/s13287-020-02122-7.

## Background

Retinitis pigmentosa (RP) is a collective term describing the range of disorders with inherited, progressive photoreceptor and/or retinal pigment epithelial cell degeneration and dysfunction [1]. The clinical manifestation typically starts with night blindness (nyctalopia), followed by the progressive loss of peripheral vision (tunnel vision), until the patients become legally blind [2], usually during the third or fourth decade of life. To date, RP has been associated with more than 100 genes [3]. Each gene plays an important function in the phototransduction cascade, ciliary transport, or visual cycle. Mutations in one of these genes might result in a lack or defect of functional protein in the cascade, eventually aborting the entire process. The mutation-dependent approach, such as gene therapy, employs viral vectors to deliver the therapeutic gene to the target cells to restore the defective gene function. However, this approach will only be effective if performed in the early stage of the disease, when the target cells are still present [4].

Recently, advances arise in the use of stem cells as treatment modalities for retinal diseases, including RP. Several stem cell-based treatments are in phase I or I/II clinical trials and registered in clinicaltrials.gov. Considering the low immunogenicity and ease of isolation and expansion, mesenchymal stem cells (MSCs) become a promising candidate for retinal cell therapy [5, 6]. Bone marrow-derived MSCs (BM-MSCs) and adipose-derived MSCs (AD-MSCs) are among the most prominent sources for retinal cell therapy trials. The primary benefit of MSCs is the paracrine protective effect toward the retinal pigment epithelium (RPE) and photoreceptors. MSCs spontaneously produce hepatocyte growth factor (HGF), nerve growth factor, and vascular endothelial growth factor, which act as tissue regeneration factors. MSCs also produce immunomodulatory cytokines, such as interleukin-6 (IL-6) and transforming growth factor-beta (TGF-β), which inhibit the inflammatory reaction of the cells during disease progression. Additionally, an interesting treatment alternative involves the stem cell-free approach [7, 8]. An in vitro study evaluating the MSC secretomes found that they possessed neuroprotective properties toward the retinal structure and neuropreservative effects delaying the photoreceptor cell degeneration [9]. These effects are promising for further development of cell-free treatment strategies to minimize the unwanted effects from stem cells. Nevertheless, further studies are required to identify the safety profiles and mechanisms of action of these secretome components.

Two independent pre-clinical studies showed that transplantation of BM-MSCs can prevent retinal vascular degeneration and prolong photoreceptor cell survival from retinal degeneration in the mouse model [10, 11]. At least three clinical trials with published results evaluated the safety of intravitreal injection of autologous bone marrow-derived stem cells. Overall, the stem cell products and delivery procedure are considered safe although a complication of fibrosis formation in the vitreous cavity had been reported [12]. Moreover, there were noticeable improvements of visual functions at 3–6 months during patient follow-up [12–14]. Taken together, safety data from up-coming clinical trials are highly necessary to verify the safety and efficacy of stem cell therapy for RP. In this study, we aimed to evaluate the safety, feasibility, and short-term efficacy of a phase I clinical trial involving the production and intravitreal injection of autologous BM-MSCs in subjects with advanced RP.

## Methods

### Study design and ethics

This prospective non-randomized open-label phase I clinical trial assessed the safety, feasibility, and short-term efficacy of an intravitreal injection of autologous mesenchymal stem cells in participants with RP. The study had been approved by the institutional review board (IRB) of the Faculty of Medicine Siriraj Hospital Mahidol University (approval number Si126/2010, dated 2 March 2010) and was registered in clinicaltrials.gov (NCT01531348). This study was conducted in Siriraj Hospital Mahidol University, Thailand, from February 2012 until March 2020. The involvement of human subjects adhered to the Declaration of Helsinki. All participants provided written informed consents prior to enrollment.

### Study participant recruitment

**Fig. 1:**
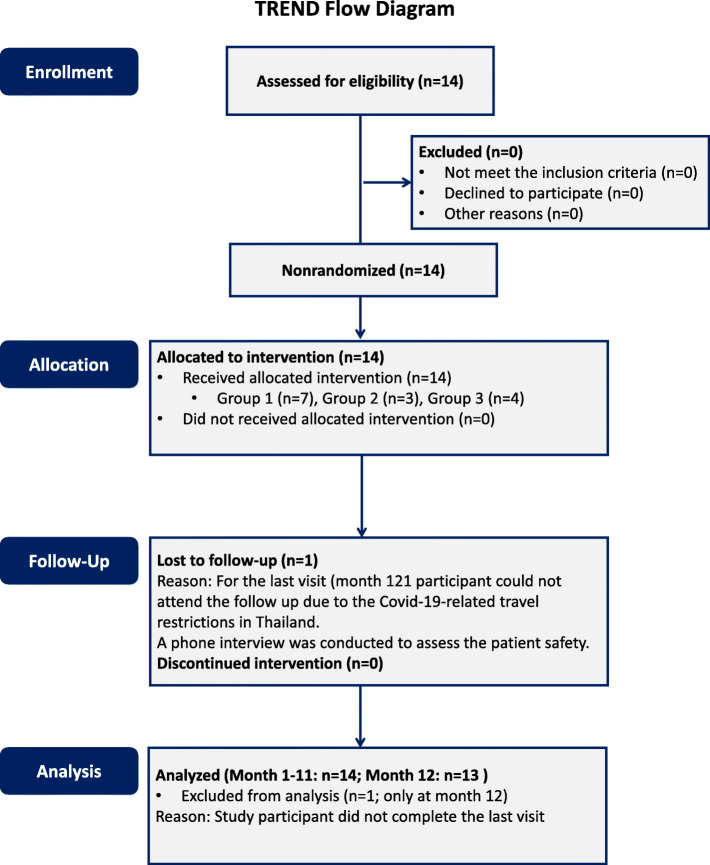
The transparent reporting of evaluations with non-randomized design (TREND) flow diagram. The TREND flow diagram describes the number of study participants during trial enrollment, allocation, follow-up, and discontinuation of intervention of the intravitreal injection of autologous bone marrow-derived mesenchymal stem cells

We enrolled 14 participants with advanced RP, as diagnosed by experienced ophthalmologists, by invitation without coercion and influence. The inclusion criteria were as follows: (1) age between 18 and 65 years old, (2) best corrected visual acuity (BCVA) of more than or equal to logarithm of minimum angle of resolution (logMAR) of 0.48, (3) central visual field (VF) less than or equal to 20°, and (4) a nonrecordable electroretinogram (ERG) or an amplitude of less than 25% of the normal value. The exclusion criteria were participants with other ophthalmologic conditions that could confound the interpretation, severe underlying diseases (including asthma, heart, liver, or renal failure), pregnancy or lactation, or failure to return for follow-up. The participants could withdraw from the enrollment at any time upon personal request. The transparent reporting of evaluations with non-randomized design (TREND) flow diagram describing the participant recruitment and enrollment processes is shown in Fig. 1.

### Isolation and characterization of bone marrow-derived mesenchymal stem cells

Approximately 20 ml of autologous bone marrow was aspirated from the posterior iliac crest in a sterile heparin-containing syringe and produced BM-MSCs under controlled condition in the cleanroom ISO 5 (class 100, Grade A). BM-MSCs were maintained in Dulbecco’s modified Eagles medium (Invitrogen, USA) supplemented with 10% fetal bovine serum (Bovogen Biologicals, Australia) and antibiotic/antimycotic (Sigma, USA), at 37 °C with 5% CO_2_. The third passage of BM-MSCs was used for intravitreal injection.

The positive expression of cluster of differentiation (CD) 73, CD90, and CD105 and the lack of expression for the surface markers CD34, CD45, and human leukocyte antigen (HLA)-DR were ensured by flow cytometry (FACSCanto II, BD Biosciences, USA). The BM-MSCs were also confirmed for their trilineage differentiation ability to adipocyte (Mesenchymal Stem Cell Adipogenesis Kit: Millipore, Germany), osteocyte (Mesenchymal Stem Cell Osteogenesis Kit: Millipore, Germany), and chondrocyte (StemMACS™ ChondroDiff Media, human: Miltenyi Biotec, Germany). We also tested the product sterility, including aerobic and anaerobic bacteria, fungi (Automate hemoculture with BD BECTEC™ PLUS+Anerobic/F medium, BD BECTEC™ PED PLUS™ /F medium, BD BECTEC™ Myco/F Lytic medium, BD, USA), mycoplasma (Venor GeM, Minerva Biolabs, Germany), and endotoxin using limulus amebocyte lysate kinetic method (PYROGENT™-5000 Kinetic Turbidimetric LAL Assay, Lonza, USA) prior to sending the product to Siriraj Hospital Mahidol University, Thailand, under a controlled temperature of 15–20 °C.

### Intravitreal injection of mesenchymal stem cell product

All participants received a single intravitreal injection of BM-MSC suspension in sterile balanced salt solution (BSS) in the right eye (study eye), while the fellow eye served as control. Two days prior to the procedure, the participants were advised to administer the antibiotic eye drops (moxifloxacin) to both eyes to prevent infection. Under sterile condition, an intravitreal injection at the superotemporal quadrant, 3.5 mm posterior to the limbus, was performed by an experienced ophthalmologist under topical anesthesia (0.5% tetracaine hydrochloride ophthalmic solution). A 30-gauge needle was used to deliver 50 μl of autologous BM-MSC suspension into the vitreous cavity. Indirect ophthalmoscopy was performed immediately after the procedure to ensure no occlusion of the central retinal artery. All participants received moxifloxacin eye drops for 7 days post-intervention.

There were three groups of intervention: group 1 receiving 1 × 10^6^ cells, group 2 (5 × 10^6^ cells), and group 3 (1 × 10^7^ cells). We assigned the first five participants to group 1. After the acute safety profile was established in the interim analysis, the next participants were categorized into groups 1 to 3, depending on the quantity of MSCs received from the culture. At the end of the study, there were seven participants in group 1, three participants in group 2, and four participants in group 3.

### Clinical evaluation

This phase I clinical trial assessed the safety, feasibility, and short-term efficacy of intravitreal injection of BM-MSCs in participants with advanced RP. Safety was assessed by evaluating immediate and short-term adverse events (AEs). The immediate AEs were recorded during 24 h after the injection. The short-term AEs and efficacy were examined daily for the first 7 days, then once weekly for up to 4 weeks, and then monthly for a period of 12 months, except for the last participant who could not attend the 12th month follow-up during the COVID-19 pandemic. Nevertheless, we conducted a phone interview with the corresponding participant. After the study period, most participants were continued to be followed, ranging from 1.5 to 7 years. The safety parameters included the intraocular pressure (IOP) (Non-contact tonometer NT-530P, Nidek Co., LTD, Japan), cells and flare in the anterior chamber (Laser flare cell meter FC-2000, Kowa Company, Japan), anterior segment examination using slit-lamp biomicroscopy, fundus evaluation using fundus photography (VX-20 Retinal camera, Kowa Company, Japan and Optos Ultra-widefield (UWF™) retinal imaging, Optos Inc., USA), fundus autofluorescence, fundus fluorescein angiography (FFA) and indocyanine green angiography (ICG) (The Spectralis® HRA+OCT, Heidelberg Engineering Inc., Germany), and subjective complaints of participants. Other evaluations comprised BCVA (logarithmic visual acuity chart), VF (Perimeter Model 12230, American Optical CO., USA), central subfield thickness (The Spectralis®, HRA+OCT, Heidelberg Engineering Inc., Germany), and electroretinography (ERG) (Veris™, Electro-Diagnostic Imaging Inc., USA), which served as both safety and short-term efficacy parameters.

### Statistical analysis

Data were presented as mean ± SD of each time point. Data of cells and flare were presented as mean of each group. VF and central subfield thickness (CST) were evaluated collectively for all participants. A paired *t* test was used to compare the means of the BCVA between the baseline and each time point and between the study and control eye in each time point. The *P* ≤ 0.05 was considered statistically significant. The statistical analysis was performed using PASW Statistics version 18.0.0.

## Results

### Characterization of bone marrow-derived mesenchymal stem cells

**Fig. 2:**
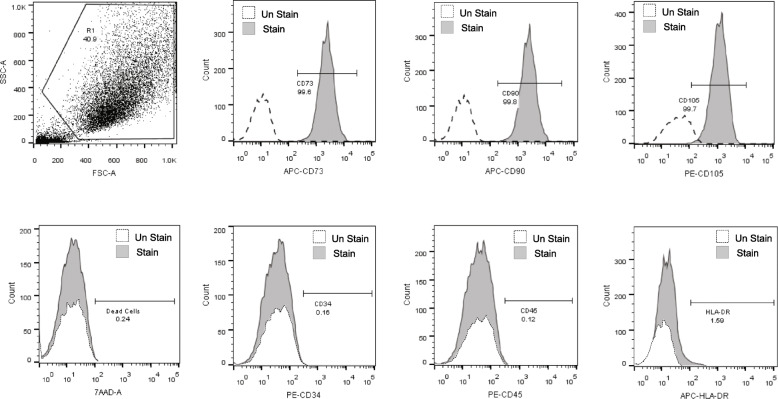
Characteristics of bone marrow-derived mesenchymal stem cells (BM-MSCs). Representative flow cytometry histogram of mesenchymal stem cell (MSC) characteristics assessed by positive expression of CD73, CD90, and CD105 and negative expression of CD34, CD45, and HLA-DR surface markers. Cell viability was assessed by the expression of the 7-amino-actinomycin D (7-AAD). Dashed lines represent the unstained control

BM-MSCs from all participants exhibited spindle-shaped-like cells. The stem cell phenotypes were in accordance with the international society for cellular therapy (ISCT), such as adherence to the plastic culture vessel; expression of more than 95% of CD73, CD90, and CD105; and negative (less than 2%) for CD34, CD45, and HLA-DR (Fig. 2). The trilineage differentiation ability to be adipocyte, osteocyte, and chondrocyte was confirmed in all samples. There was no microorganism or endotoxin contamination. The average cell viability was 92.12 ± 3.5% (Table 1).

**Table 1:** Demographic data

Study group	Age/gender/study eye	Dose (cells)	Cell viability (%)	Eye	IOL	BCVA (logMAR)	Visual field (degrees)				ERG	VEP	
							S	N	I	T		Amplitude (mV)	Latency (ms)
1	38/M/OD	1 × 10^6^	86.00	OD OS	No No	2.00 2.00	10 10	10 10	10 10	10 10	NR NR	5.465 5.985	N/A
1	58/M/OD	1 × 10^6^	87.5	OD OS	Yes Yes	1.40 1.51	10 10	10 10	10 10	10 10	NR NR	6.357 1.950	98 106
1	58/M/OD	1 × 10^6^	95.70	OD OS	No No	1.08 1.30	10 10	10 11	10 10	12 10	NR NR	2.995 5.725	134 134
1	56/F/OD	1 × 10^6^	93.00	OD OS	No No	2.30 2.30	10 25	10 10	15 40	10 24	NR NR	5.075 6.505	152 145
1	42/M/OD	1 × 10^6^	94.00	OD OS	No No	2.30 2.30	10 10	10 10	10 10	10 10	NR NR	4.615 5.550	105 98
1	46/F/OD	1 × 10^6^	94.05	OD OS	Yes Yes	2.30 2.30	N/A	N/A	N/A	N/A	NR NR	5.335 5.465	123 100
1	35/F/OD	1 × 10^6^	86.60	OD OS	No No	1.47 1.36	45 55	55 50	70 60	75 72	NR NR	14.84 11.72	120 119
2	61/M/OD	5 × 10^6^	96.00	OD OS	Yes No	1.22 1.08	10 10	10 10	10 10	10 10	NR NR	4.615 3.645	114 105
2	42/M/OD	5 × 10^6^	96.73	OD OS	No No	2.30 2.30	N/A	N/A	N/A	N/A	NR NR	4.680 3.900	106 107
2	48/M/OD	5 × 10^6^	93.75	OD OS	No No	2.70 2.70	N/A	N/A	N/A	N/A	NR NR	3.380 2.990	135 121
3	45/F/OD	1 × 10^7^	93.07	OD OS	Yes Yes	2.30 2.30	N/A	N/A	N/A	N/A	NR NR	3.510 5.335	131 135
3	37/M/OD	1 × 10^7^	92.58	OD OS	No No	2.70 2.30	N/A	N/A	N/A	N/A	NR NR	2.470 4.290	130 128
3	49/M/OD	1 × 10^7^	89.14	OD OS	Yes Yes	1.34 2.30	N/A	N/A	N/A	N/A	NR NR	N/A	N/A
3	32/F/OD	1 × 10^7^	91.60	OD OS	No No	2.00 2.00	10 10	10 10	10 10	10 10	NR NR	N/A	N/A

*BCVA* best corrected visual acuity, *ERG* electroretinogram, *F* female, *M* male, *logMAR* logarithm minimum angle of resolution, *ms* millisecond, *mV* millivolt, *IOL* intraocular lens, *OD* oculus dexter, *OS* oculus sinister, *VEP* visual evoked potential, *S* superior, *N* nasal, *I* inferior, *T* temporal, and *NR* nonrecordable

### Baseline characteristics of study participants

We recruited 14 participants with advanced RP, consisting of nine males and five females, with ages ranging from 31 to 61 (mean ± SD, 46.2 ± 9.3) years. Four participants had the intraocular lens (IOL) in both eyes and one had IOL in the study eye (Table 1), all of which underwent the surgeries longer than 6 months prior to enrollment. The average baseline BCVA was 2.00 ± 0.14 logMAR in the study eye. At baseline, the mean number of the cells and the values of flare in the anterior chamber of the study eye were 1.55 ± 0.88 cells per 0.5 mm^3^ and 6.8 ± 3.05 photons per millisecond (ph/ms), respectively, with an average IOP of 13.11 ± 1.71 mmHg. The ERG was nonrecordable in all participants. Seven out of 14 participants had VF less than 20° in the study eye, and one participant presented with a central scotoma of 20° with normal peripheral VF. In the remaining six participants, we could not evaluate the VF due to poor fixation. Seven participants were categorized based on the number of MSCs injected to group 1 (1 × 10^6^ cells), three participants to group 2 (5 × 10^6^ cells), and four participants to group 3 (1 × 10^7^ cells) (Table 1).

### Clinical safety evaluation

We did not observe any serious AEs after the intravitreal injection, such as central retinal artery occlusion, leakage, hemorrhage, retinal detachment, or endophthalmitis, in all participants. We noticed an increase in IOP in all groups (4.40 ± 2.07 mmHg in group 1; 6 ± 4.24 mmHg in group 2; and 9 ± 0 mmHg in group 3) 1 h post-intravitreal injection. However, the IOP returned to the baseline values on the first day (D1) and remained stable throughout the course of the study.

**Fig. 3:**
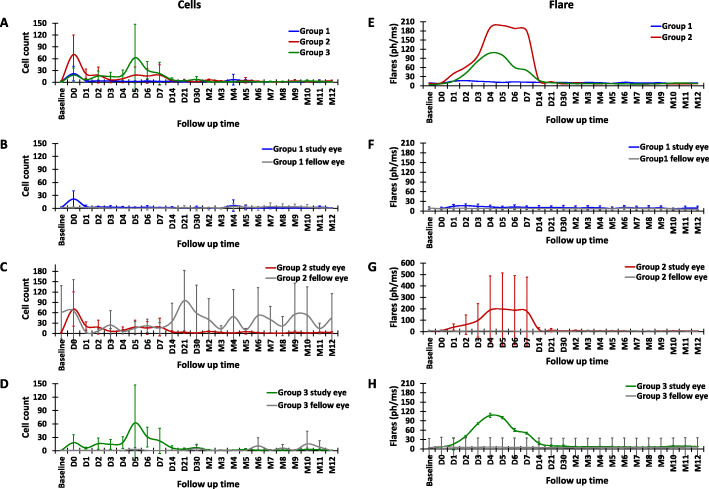
The cell numbers and flare values after the intravitreal injection of BM-MSCs. The cells and flare were evaluated daily for the first week, weekly for the first month, then monthly for the next 12 months. The graphs show the comparison of cells (**a**) and flare (**b**) in the study eyes among groups (blue, red, and green lines represent groups 1, 2, and 3, respectively). The comparison between the fellow eye (gray line) and study eye in each group (**c** and **d** for cells and flare of group 1, *N* = 7; **e** and **f** for group 2, *N* = 3; and **g** and **h** for group 3, *N* = 4). Error bars indicate the SD of cells and flare value in each group

The Kowa FC-2000 was used to quantify the flare and cells. The intensity of flare and peaks of light scattered from the particles in the aqueous reflect the inflammation and cells in the anterior chamber, respectively. It is important to note that any large particles in the aqueous that can produce peaks, such as blood cells, pigment, debris, or other cells, cannot be differentiated from inflammatory cells [15, 16]. In the present study, the number of cells in the anterior chamber increased and peaked immediately in the study eye after the injection in groups 1 and 2 (Fig. 3a, c, e). In group 3, the cells gradually increased and reached the peak at D5 (Fig. 3a, g). Compared among the three groups, the highest increase in cells from the baseline was observed in group 2. In group 1, which received the lowest number of BM-MSCs, the cells returned to the baseline after D1, while in groups 2 and 3 they returned to the baseline after D14. The protein flare values in group 1 did not show a remarkable increase after the injection (Fig. 3d), while those in groups 2 and 3 peaked at D4–5 (Fig. 3f, g). Similar to the cells, the highest increase in flare values from the baseline was found in group 2. This was due to the acute inflammation in one participant in group 2. After given eye drops containing tobramycin and dexamethasone, and 1% atropine eye drops, the inflammation subsided within 1 week. In groups 2 and 3, the flare values returned to the baseline after 14 days and remained stable throughout the course of the study. The cells and flare in the fellow eyes remained stable, except in group 2 (Fig. 3e), since two participants displayed fluctuating high numbers of cells in the fellow eye throughout the study.

**Fig. 4:**
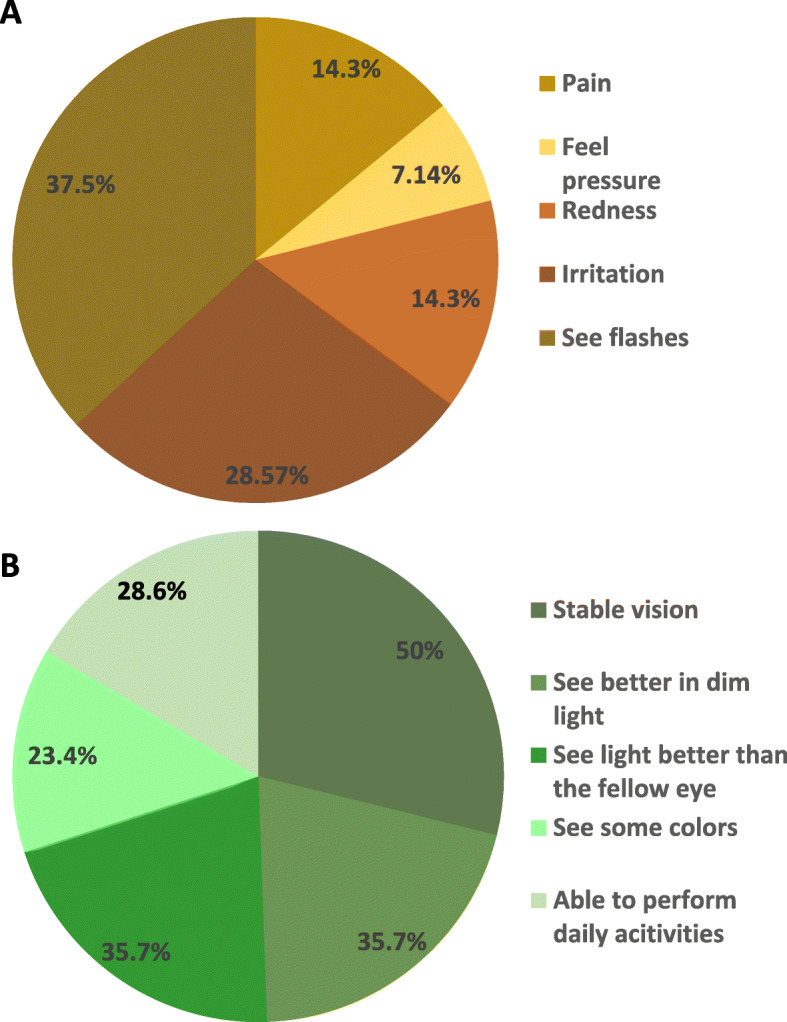
Subjective complain during the study. The subjective complain for the safety (**a**) and efficacy (**b**) of autologous intravitreal injection of BM-MSCs during the 12-month follow-up

During the first week post-injection, two participants (14.28%) complained of mild pain in the study eye, one (7.14%) reported feeling pressure, two (14.28%) with redness, and four with mild irritation in the study eye. All symptoms subsided spontaneously within 1 week without treatment. Five participants (35.71%) described seeing flashing or moving lights or dots occasionally. No participant reported extreme discomfort in the study eye (Fig. 4).

**Fig. 5:**
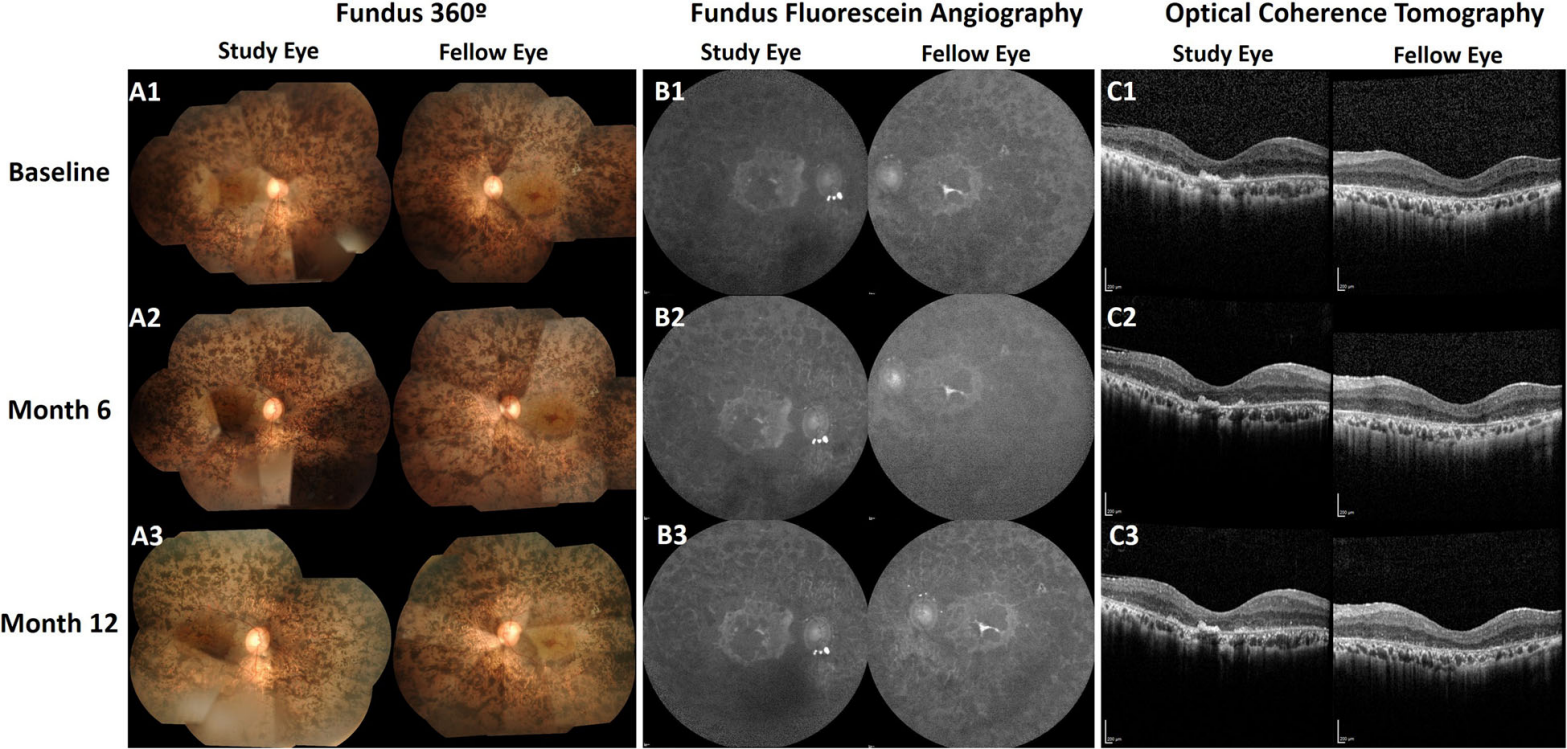
Retinal appearances during the study period. **A1** Fundus montages demonstrate generalized pigmentary changes with heavy bone spicules and macular involvement. **A2** and **A3** images, representing months 6 and 12, respectively, show similar appearances. **B1** Fundus autofluorescence images describe the central hyper-autofluorescence surrounding the island of hypo-autofluorescence in the macula, where autofluorescence indicates the presence of normal retinal pigment epithelium (RPE). **B2** and **B3** images represent months 6 and 12, respectively, with no remarkable changes. **C1**, **C2**, **C3** The optical coherence tomography indicates central subfield thickness at baseline, month 6, and month 12, respectively. The study eye and fellow eye show similar appearances. Images were retrieved from a study participant in group 1

In the course of 12 months, no participant experienced a sudden decline in the BCVA or VF. The fundus photographs, fundus autofluorescence, FFA, ICG (data not shown), and the OCT showed no significant changes in pigment accumulation, areas of hypo- and hyper-autofluorescence, the appearances of retinal and choroidal vasculature, and central subfield thickness, respectively, in the study eye and the fellow eye. This indicated the stable condition of RP, with no remarkable deterioration of the disease (Fig. 5).

**Fig. 6:**
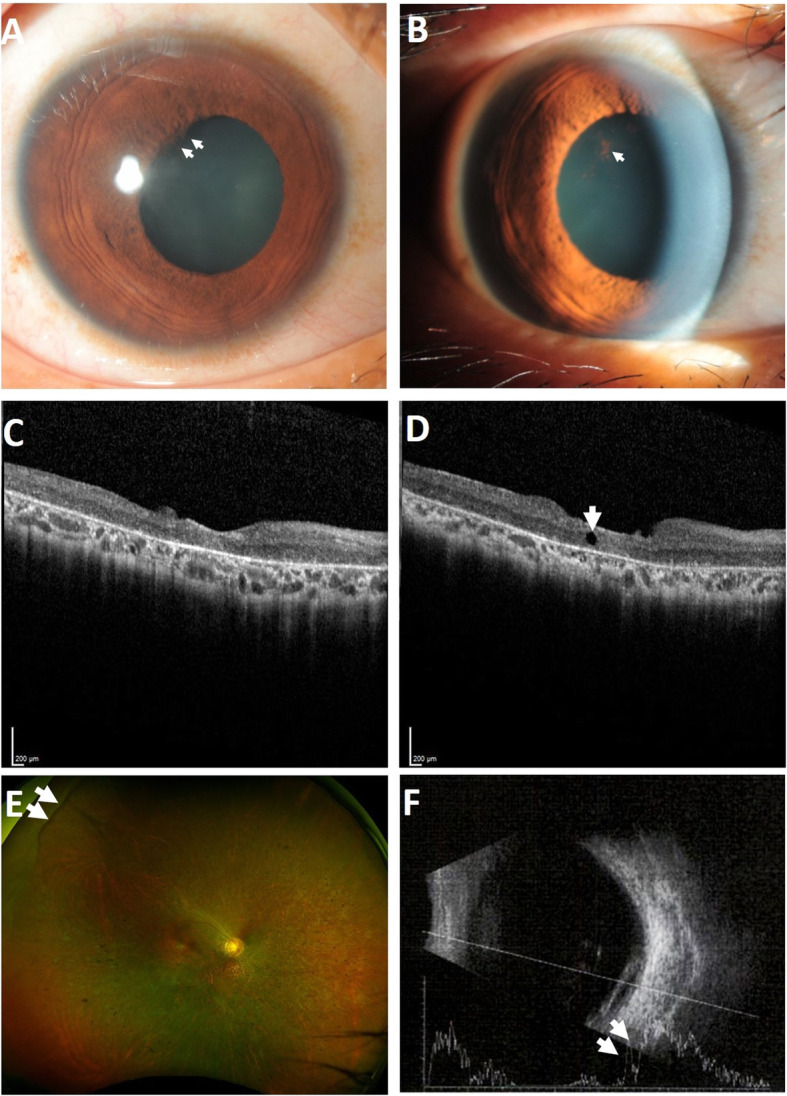
Adverse events in the study participants receiving intravitreal injection of BM-MSCs. **a** Slit-lamp biomicroscopy of a study participant with posterior synechiae (indicated by arrows). **b** After treatment with the cycloplegic agent, the posterior synechiae were released, leaving some residual pigments adhered on the lens surface (indicated by the arrow). **c** Optical coherence tomography imaging in a study participant at baseline and **d** at month 3 with mild cystoid macular edema (indicated by the arrow). **e** The ultra-widefield retinal imaging shows choroidal detachment (indicated by arrows) at the superotemporal periphery. **f** Ultrasound imaging demonstrates double-peak reflectivity (indicated by arrows) with anechoic suprachoroidal space

We observed several mild AEs, none of which affected the BCVA in the study eye. One participant in group 3 developed localized posterior synechiae at the superotemporal quadrant, at D6 (Fig. 6a). After treatment with 1% atropine eye drops, the synechiae was released at D14 (Fig. 6b). Minimal cystoid macular edema (CME) occurred in one participant in group 1 at month (M) 3 and persisted until the last follow-up at year (Y) 2.5 (Fig. 6c, d). We noticed a slight displacement of the IOL toward the temporal side of the study eye, with the IOL optic still in the visual axis, in two out of five participants with IOL, from groups 2 and 3, which started at M8 and M6, respectively (photographs not shown). The last mild AE was observed in a participant in group 1 who demonstrated localized flat choroidal detachment at the superotemporal quadrant of the study eye at M9. The ultra-widefield retinal imaging displayed that the choroidal detachment was slightly elevated at Y5 (Fig. 6e). The ultrasound demonstrated double-peak reflectivity in the A-scan with anechoic suprachoroidal space in the B-scan, indicating fluid accumulation (Fig. 6f).

**Fig. 7:**
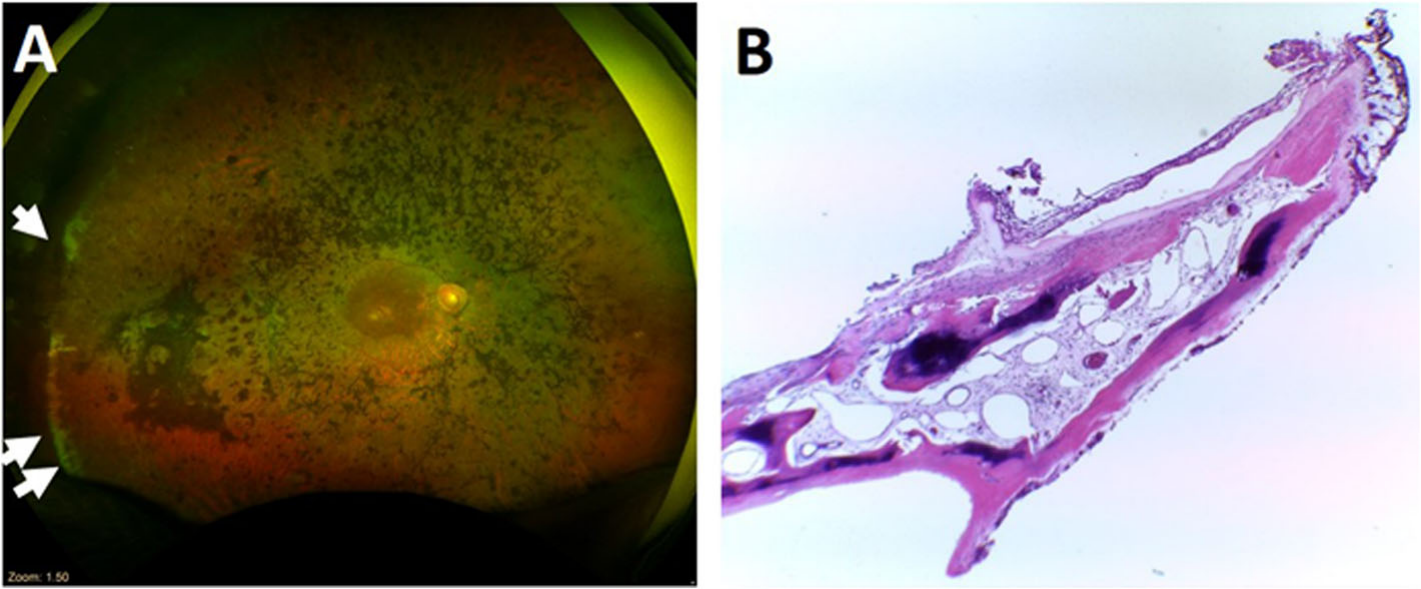
Ultra-widefield retinal image and histopathological finding of a participant with a severe adverse event. **a** The ultra-widefield retinal image displays the margin of retinectomy upon removing the fibrous membrane along the vitreous base temporal periphery (indicated by arrows). **b** Histopathological section of the membrane shows osseous metaplasia or heterotopic ossification (hematoxylin and eosin (HE) staining, magnification × 40)

Although this phase I study was completed in the 12-month period, we constantly monitored 12 out of 14 study participants during hospital visits, with a duration ranging from 1.5 to 7 years (mean ± SD, 4 ± 1.8 years). We documented two AEs. The first one happened at Y3M4, when a participant from group 2 experienced sudden vision loss in the study eye after waking up in the morning. Upon immediate evaluation, we found diffuse vitreous hemorrhage, which obscured fundus details. Pars plana vitrectomy was performed to remove the vitreous hemorrhage (Fig. 7a). Intraoperatively, we observed thick fibrous membrane along the vitreous base at the superotemporal quadrant. The fibrous membrane wrapped around the capsular bag; thus, the IOL and the capsular bag had to be removed. This membrane also caused traction to the peripheral retina with retinal dialysis superotemporally. Histopathological evaluation of the membrane demonstrated a membranous sheet of bone surrounded by ciliary tissue. Higher magnification showed calcified matrix sheets with parallel alignment and regularly distributed osteocytes, indicating osseous metaplasia or osseous heterotopia in the ciliary body (Fig. 7b). After the operation, the retina was attached and the BCVA was restored to the same level as the time of enrollment (logMAR 1.22 and 1.20, at baseline and Y5, respectively). Another mild AE happened in one participant from group 1, who developed minimal IOL subluxation in both eyes at Y4 post BM-MSC injection.

### Short-term efficacy evaluation

In this study, we evaluated the visual acuity, visual field, and central subfield thickness for the short-term efficacy of the BM-MSCs. The BCVA was evaluated individually for each group. Since the number of participants that could perform reliable VF and CST assessment was low in some groups, we analyzed the available data as a mean from all participants, comparing between the baseline and each time point.

**Fig. 8:**
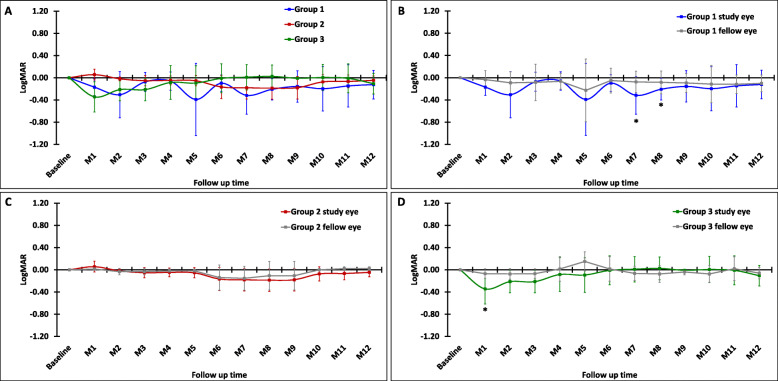
Best corrected visual acuity (BCVA) after the intravitreal BM-MSC injection. The changes in visual acuity (logMAR) of each month were normalized to the baseline. **a** The comparison of the BCVA in the study eye among groups (blue, red, and green lines indicate groups 1, 2, and 3, respectively). The comparison of the BCVA between the study eye (gray line) and fellow eye of groups 1 (**b**), 2 (**c**), and 3 (**d**). Data represent mean of study participants from each group; group 1: *N* = 7, group 2: *N* = 3, and group 3: *N* = 4 (except for M12: *N* = 3). Error bars indicate the SD of cells and flare value in each group. Asterisks indicate the statistical significance at *P* value < 0.05

There was a slight improvement of BCVA in all study groups; however, it slowly returned to the baseline within 12 months (Fig. 8a). Interestingly, when compared to the fellow eye, the highest improvement was observed in group 1 (Fig. 8b), which received the lowest number of BM-MSCs. Specifically, when compared to the baseline, the BCVA in group 1 improved at M2, 5, 7, and 8, reaching statistically significant improvements at M7 (*P* = 0.04) and M8 (*P* = 0.02), slowly regressed after M9, and reached the baseline at M12. In group 2, the BCVA improvement started after M5 and persisted up to M9, then slowly declined until it reached the baseline at M12 (Fig. 8c). Conversely, in group 3, although the BCVA significantly improved immediately at M1 (*P* = 0.04), it returned to the baseline at M6 (Fig. 8d). We noted that both the study and fellow eyes showed the similar trends of the BCVA changes, especially in group 1 and group 2, although the study eye showed higher improvements. A pair match with the fellow eye was also tested and we did not observe a statistically significant difference in the BCVA between the study and the fellow eye, although some participants reported subjective improvements of vision on activities of daily living. The details of logMAR values (mean ± SD) of each study group were provided in Supplementary Table S1.

Eight out of 14 participants showed unremarkable VF changes between the baseline and at M12, while it was not able to be evaluated in the remaining six participants due to severe reduction in the visual acuity. We evaluated the CST in 11 out of 14 participants (the CST was unmeasurable in the remaining three study participants). The average CST at the baseline was 172 ± 59.2 μm in the study eyes and remained stable throughout the study, i.e., M1 (178 ± 61.7 μm), M3 (178 ± 58.7 μm), M6 (176 ± 60.1 μm), and M12 (176 ± 59.8 μm). On the other hand, the fellow eyes at M12 displayed thinner CST than baseline (185 ± 66.8 vs 178 ± 65.4 μm). Interestingly, we noticed slight improvements in individual CST in the study eye in three out of 11 participants (27.27%), with baseline/M12 values of 142/150 μm, 150/177 μm, and 102/131 μm, respectively. The BCVA of two participants (with CST baseline/M12 of 142/150 μm and 150/177 μm) showed some improvement in the study eye (logMAR 1.34 at baseline to 1.02 at M12; and logMAR 2.30 at baseline to 1.66 at M12, respectively). However, this did not occur in the other participant with CST improvement.

Subjectively, most participants experienced improvements in the quality of life during the 12-month period after the BM-MSC injection. Seven out of 14 (50%) participants described a stable vision, five (35.7%) participants could see better in dim light, five (35.7%) could see light better than the fellow eye, three (21.4%) could see some colors, and four (28.6%) could perform daily activities better, such as walking to the bathroom at night, using the cell phone, watching TV, and riding the bicycle (Fig. 4).

## Discussion

Herein, we demonstrated the safety, feasibility, and short-term efficacy study of intravitreal injection of autologous BM-MSCs in participants with advanced RP. To date, this is the first clinical trial using BM-MSCs in RP in Thailand with one of the longest durations of participant monitoring.

Several studies intensively assessed the safety profiles of several stem cell types for retinal disease cell-based therapy, including hematopoietic stem cells [14], retinal progenitor cells (RPCs) [17], embryonic stem cells (ESCs) [18], induced pluripotent stem cells (iPSCs) [19], and MSCs [11, 12]. In the present study, the autologous BM-MSCs were selected. Since the MSCs exhibit the lower potential of differentiation compared to RPCs, ESCs, and iPSCs, this stem cell type has a lower risk of differentiating into undesired tissues, teratoma formation, immune rejection (even from allogeneic sources), and ethical concerns to its use [20, 21]. More importantly, many studies demonstrated the promising potential of the MSCs for retinal disease therapy. Among other hypotheses, the paracrine effects, including tissue regeneration and immunomodulatory factors, are well-accepted benefits of the MSCs [7, 8, 22]. Theoretically, these secreted soluble factors could slow the degeneration of the neuroretinal cells with the possibility of restoring their functions [20]. More recently, the stem cell-free approach using the secretory components from MSCs, including the soluble mediators and extracellular vesicles (exosomes and microvesicles), are under investigation [9, 23].

The source of MSCs and delivery route are fundamental clinician decisions. Among those MSCs, BM-MSC is the most commonly used, with the longest track record, source of human MSCs [24]. In this study, we utilized the autologous BM-MSCs to avert the host-versus-graft rejection which might occur after an allogeneic transplantation. However, recently, remarkable advancements were reported in the use of allogeneic transplantation of Wharton’s jelly-derived MSCs, which showed promising safety and efficacy results in clinical trials as a candidate cell therapy for retinitis pigmentosa [25, 26]. There are several administration routes of MSCs that had been validated. Intravenous is among the easiest and the least invasive route. This method is most common for MSC delivery. However, this route requires a large number of MSCs since MSCs could circulate to various organs and be trapped within the small capillaries [27]. Therefore, this route might not be suitable for retinitis pigmentosa. Subretinal transplantation would deliver the cells directly under the retina in an immunoprivileged site, but the procedure is more complex and invasive. Moreover, this route is restricted with the number and volume of MSC product. Intravitreal transplantation of MSCs could overcome the volume restriction and the procedure is less invasive. The MSCs administered via this route hardly pass the internal limiting membrane of the retina [28], but the secreted paracrine factors from MSCs could [29]. Recently, a phase 3 clinical study reported the superior safety and efficacy profile of subtenon MSC injection since this cavity is hypovascular and the secreted growth factors could pass through the choroid to the subretinal space [25].

The primary objective of this study was to assess the safety of the intravitreal injection of BM-MSCs. After the injection, participants experienced an increase in cells and flare in the anterior chamber, indicating the occurrence of inflammation. The mildest inflammation happened in group 1, which received the lowest number of cells. However, this inflammatory reaction was transient and all values returned to the baseline by 14 days in all groups. It is acceptable that the IOP might increase immediately after an intravitreal injection. Although there were no significant differences among the study groups, we observed the largest mean difference of IOP (1 h after the injection vs baseline) in group 3, which received the highest number of BM-MSCs. Nonetheless, the IOP in all groups returned to the baseline within 1 day. Throughout the study period, no participant experienced a rapid decline in the visual acuity or visual field. Fundus evaluation also revealed stable features of the disease. These results indicated the safety of the procedure and non-toxicity of the stem cell products used in our study toward the RPE and photoreceptors.

We noticed a statistically significant improvement of the mean BCVA compared to baseline in group 1 at M7–8 and in group 3 at M1. Although there were no significant changes in group 2, we noted the BCVA improvement during M6–8. This effect was transitory and the BCVA returned to the baseline at M12. We managed to follow 12 participants beyond the study period and discovered that the BCVA relatively declined with time (data not shown). These indicated that BM-MSCs might help to slow down the disease progression in a certain period of time. The re-injection of BM-MSCs or an alternative substance, such as exosomes, should be further evaluated.

Interestingly, we observed that the fellow eye of participants in groups 1 and 2 showed the same trend of improvements in the BCVA as the study eye, although to a lesser extent. This phenomenon should also be explored to elucidate whether the effects of the secreted factors from stem cells also benefit the fellow eye. Since the primary benefit of stem cells is to maintain the longevity of the cells, in the future, it might be advantageous to explore the efficacy in participants with less advanced disease.

We found a localized serous choroidal detachment at M9 in a participant from group 1. Since this participant had stable IOP, plausible explanation includes trauma or inflammation from the injection, which led to fluid accumulation under the choroid. Although we noted a slight increase in the thickness of the choroidal detachment, it remained asymptomatic, required no intervention, and did not affect the BCVA.

Our study pointed out the high rate of IOL displacement (60%) after the intravitreal injection of BM-MSCs. Since in all cases the displacement was directed toward the temporal side, it was thought to be caused by the traction force to the zonules and lens capsule near the injection site. However, one of these three participants had IOL displacement in both the study and fellow eyes. Therefore, the findings in this participant might be related to the RP condition itself rather than the BM-MSC injection. Interestingly, we did not observe any lens subluxation in those participants with crystalline lenses. RP is known to increase the risk of IOL subluxation due to zonular insufficiency or weakness [30, 31]. The intravitreal injection procedure might aggravate the weakness of the zonules or cause fibrosis formation, resulting in traction on the zonules and lens capsule [32]. Since the capsule after IOL insertion is more pliable than that of the crystalline lens, this might cause the IOL to be pulled easier. It is important to evaluate the lens position prior to the intervention.

After the 12-month period, we observed severe AE in one participant (8.3%), who developed osseous metaplasia along the vitreous base. However, after surgical treatment, the retina was attached and the BCVA returned to the same level as prior to the injection. A previous study reported intraocular ossification occurring in 4.8 to 18% in the enucleated eye cases [33, 34]. Possible causes include retinal detachment, chronic inflammation, trauma, presence of bone morphogenic proteins, calcification of drusen, and the differentiation or paracrine effects of mesenchymal stem cells [35, 36]. This participant experienced a slight displacement of the IOL to the temporal side at M8 after the BM-MSC injection, which was probably attributed to the fibrous formation near the injection site. Chronic inflammation, which underlies the pathogenesis of RP progression, involves the secretion of cytokines and chemokines, such as monocyte chemotactic protein 1 (MCP1), into the vitreous [37]. These proinflammatory cytokines might support the trans-differentiation of fibroblast into osteoblast, thereby causing osseous metaplasia in the vitreous. The MCP1 is also known to be involved in recruiting inflammatory cells and increasing fibroblasts. Another plausible explanation is the paracrine activities of the BM-MSCs, which might stimulate osteogenic differentiation of fibroblasts, rather than the differentiation of the MSCs into osteocytes themselves [38]. Future studies warrant the thorough evaluation of early signs of fibrosis formation following intravitreal injection of MSCs.

We analyzed the correlation between the three different doses of BM-MSCs assigned to group 1 (1 × 10^6^ cells/eye), group 2 (5 × 10^6^ cells/eye), and group 3 (1 × 10^7^ cells/eye), and the occurrence of AEs. We found no direct correlation between the increased number or severity of AEs and the higher dose of the BM-MSCs. Mild AEs, such as flat choroidal detachment and minimal CME, and posterior synechiae happened in participants in groups 1 and 3, respectively. Slight IOL displacement occurred in one participant in all groups. Osseous metaplasia, which was the only serious AE in this study, arose in a participant in group 2. Nevertheless, the levels of IOP, flare, and cells indicated that group 1 showed the most favorable short-term safety profile.

At least four studies attempted to evaluate the safety or efficacy profiles of the intravitreal injection of bone marrow-derived stem cells in the retinal diseases including RP (Supplementary Table S2, available online) [12–14, 39]. Most of the phase I studies enrolled smaller number of participants. We recruited 14 participants in a span of 8 years. In the early phase of the study, we enrolled the participant one by one to ensure that the intervention was safe prior to escalating the doses of the cells. Most participants who completed the 12-month period continued to visit our institute and were continually monitored. We found two AEs after M12, which became our consideration for the next phase of the study, although it was difficult to exactly determine whether these AEs were caused by the intervention, the BM-MSC product, or the RP disease progression. Since our study employed larger numbers of participants with advanced RP and had longer follow-up time, we observed several AEs that were not reported in earlier clinical studies. To minimize the AEs that could appear in future clinical trials, several points need to be carefully considered. First, patient recruitment should be performed with particular assessments of the lens position and the zonular support. Second, the quality control standards of the stem cell products must be maintained. Lastly, participant follow-up should include a thorough examination of the peripheral retina using ultra-widefield OCT to detect early changes in that area. In the future, a cell-free therapy approach could be considered. Nevertheless, we provided the evidence of the feasibility of the intervention as well as the longer-term safety of the intravitreal injection of autologous BM-MSCs for RP.

## Conclusion

All things considered, intravitreal injection of autologous BM-MSCs showed good safety profiles, with several mild adverse events, including slight IOL displacement and flat choroidal detachment. One participant with a serious adverse event was successfully managed surgically. We established the short-term efficacy, reflected by stable conditions and slow disease progression. It is noteworthy that some participants reported the improvement in the quality of life after receiving this intervention. Among the three different doses of BM-MSCs, the lowest dose showed the most promising safety and short-term efficacy profiles. Therefore, in future studies, autologous BM-MSC injection with the dose of 1 × 10^6^ cells per eye will be explored in participants with less advanced RP to provide a clearer efficacy profile of this intervention. Most importantly, to minimize the risk of AEs that could happen in the future, the quality control of the stem cell product, participant follow-up, and comprehensive ophthalmological evaluation will be exclusively performed.

## Supplementary Information

**Additional file 1: S1 Table.** Best corrected visual acuity (logMAR).

**Additional file 2: S2 Table.** Comparison of autologous bone marrow-derived stem cells in clinical trials.

## Data Availability

The datasets used and/or analyzed during the current study are available from the corresponding author upon request.

## Abbreviations

7-AAD: 7-Amino-actinomycin D
AD-MSCs: Adipose-derived mesenchymal stem cells
AE: Adverse event
BCVA: Best corrected visual acuity
BM-MSCs: Bone marrow-derived mesenchymal stem cells
CME: Cystoid macular edema
CST: Central subfield thickness
ERG: Electroretinogram
ESCs: Embryonic stem cells
FFA: Fundus fluorescein angiography
FSC-A: Forward scatter area
HE: Hematoxylin and eosin
ICG: Indocyanine green angiography
IOL: Intraocular lens
IOP: Intraocular pressure
IRB: Institutional review board
iPSCs: Induced pluripotent stem cells
ISCT: International Society for Cellular Therapy
logMAR: Logarithm of minimum angle of resolution
MSCs: Mesenchymal stem cells
ph/ms: Photons per millisecond
RP: Retinitis pigmentosa
RPCs: Retinal progenitor cells
RPE: Retinal pigment epithelium
SSC-A: Side scatter area
TREND: The transparent reporting of evaluations with non-randomized design
VF: Visual field
