# Supplementary material for: Intravitreal autologous mesenchymal stem cell transplantation: a non-randomized phase I clinical trial in patients with retinitis pigmentosa
Source: Stem Cell Res Ther. 2021 Jan 9;12:52. doi: 10.1186/s13287-020-02122-7 (PMC7796606; doi:10.1186/s13287-020-02122-7)
Supplement: Supplementary file 1 — Additional file 1: S1 Table. Best corrected visual acuity (logMAR). [file 13287_2020_2122_MOESM1_ESM.docx]

**S1 Table. Best corrected visual acuity (logMAR)**

| **Study**  **group** | **Eye** | **BCVA logMAR (mean ± SD)**  **Follow-up time** | | | | | | | | | | | | |
| --- | --- | --- | --- | --- | --- | --- | --- | --- | --- | --- | --- | --- | --- | --- |
|  |  | **Baseline** | **M1** | **M2** | **M3** | **M4** | **M5** | **M6** | **M7** | **M8** | **M9** | **M10** | **M11** | **M12** |
| 1 | study eye | 1.84 ± 0.51 | 1.64 ± 0.53 | 1.98 ± 0.42 | 1.74 ± 0.54 | 1.78 ± 0.50 | 1.44 ± 0.52 | 1.71 ± 0.49 | 1.49 ± 0.50 | 1.50 ± 0.37 | 1.65 ± 0.45 | 1.61 ± 0.43 | 1.66 ± 0.51 | 1.71 ± 0.41 |
|  | fellow eye | 1.69 ± 0.44 | 1.71 ± 0.45 | 1.68 ± 0.50 | 1.65 ± 0.43 | 1.68 ± 0.48 | 1.51 ± 0.58 | 1.74 ± 0.44 | 1.66 ± 0.47 | 1.59 ± 0.42 | 1.67 ± 0.54 | 1.64 ± 0.45 | 1.64 ± 0.45 | 1.64 ± 0.44 |
| **2** | study eye | 2.10 ± 0.72 | 2.16 ± 0.63 | 2.08 ± 0.75 | 2.05 ± 0.81 | 2.05 ± 0.80 | 2.05 ± 0.81 | 1.93 ± 0.64 | 1.92 ± 0.66 | 1.91 ± 0.67 | 1.92 ± 0.66 | 2.03 ± 0.84 | 2.03 ± 0.83 | 2.05 ± 0.80 |
|  | fellow eye | 2.03 ± 0.84 | 2.04 ± 0.82 | 1.99 ± 0.90 | 1.99 ± 0.90 | 2.01 ± 0.88 | 2.01 ± 0.88 | 1.89 ± 0.72 | 1.87 ± 0.74 | 1.92 ± 0.66 | 1.92 ± 0.66 | 2.03 ± 0.84 | 2.05 ± 0.81 | 2.05 ± 0.81 |
| **3** | study eye | 2.09 ± 0.57 | 1.74 ± 0.66 | 1.90 ± 0.69 | 1.90 ± 0.69 | 2.00 ± 0.60 | 1.99 ± 0.62 | 2.08 ± 0.74 | 2.10 ± 0.70 | 2.11 ± 0.67 | 2.08 ± 0.23 | 2.09 ± 0.71 | 2.08 ± 0.74 | 2.01 ± 0.88 |
|  | fellow eye | 1.98 ± 0.47 | 1.91 ± 0.56 | 1.90 ± 0.57 | 1.91 ± 0.56 | 1.99 ± 0.62 | 2.13 ± 0.35 | 1.99 ± 0.62 | 1.91 ± 0.60 | 1.90 ± 0.62 | 1.94 ± 0.55 | 1.90 ± 0.62 | 2.00 ± 0.61 | 1.91 ± 0.68 |

BCVA: Best corrected visual acuity, logMAR: logarithm of minimum angle resolution; M: month; SD: standard deviation
