# Supplementary material for: Intravitreal autologous mesenchymal stem cell transplantation: a non-randomized phase I clinical trial in patients with retinitis pigmentosa
Source: Stem Cell Res Ther. 2021 Jan 9;12:52. doi: 10.1186/s13287-020-02122-7 (PMC7796606; doi:10.1186/s13287-020-02122-7)
Supplement: Supplementary file 2 — Additional file 2: S2 Table. Comparison of autologous bone marrow-derived stem cells in clinical trials. [file 13287_2020_2122_MOESM2_ESM.docx]

**S2 Table. Comparison of autologous bone marrow-derived stem cells in clinical trials**

| **Study** | **Siqueira et al., 2011** | **Siqueira et al., 2015** | **Park et al., 2015** | **Satarian et al., 2017** | **This study** |
| --- | --- | --- | --- | --- | --- |
| **ClinicalTrial.gov** | NCT01068561 | NCT01560715 | NCT01736059 | N/A | NCT01531348 |
| **Clinical trial phase** | Phase I | Phase II | Phase I | Phase I | Phase I |
| **Country** | Brazil | Brazil | USA | Iran | Thailand |
| **Number of participants** | 5 | 20 | 6 | 3 | 14^a^ |
| **Follow up period (months)** | 10 | 12 | 6 | 12 | 12  (monitor up to 1.5-7 years) |
| **Disease(s)** | RP and  cone-rod dystrophy | RP | CRAO/CRVO,  Stargardt,  AMD, and RP | RP | RP |
| **Stem cell type** | Autologous  BM-derived hematopoietic (CD34+) stem cells | Autologous  BM-derived hematopoietic (CD34+) stem cells | Autologous  BM-derived CD34^+^ cells | Autologous  BM-MSCs  CD34^low/-^ | Autologous  BM-MSCs  CD34^-^ |
| **Number of cells** | 1.68 x 10^4^ | 1.68 x 10^4^ | 1-7 x 10^6^ | 1 x 10^6^ | 1-10 x 10^6^ |
| **Route of injection** | intravitreal | intravitreal | intravitreal | intravitreal | Intravitreal |
| **Safety** | -1/5 participant: Subconjunctival hemorrhage  - No other important AE | No important AE | - 1/6 participants: mild extrafoveal enlargement of geographic atrophy | - 1/3 participants: extensive pre-retinal and vitreous fibrosis | - 1/14 participants: Posterior synechiae (D6)  - 1/14 participants: mild CME (M3)  - 3/5 participants: IOL displacement (M6, M8, and Y4)  - 1/14 participants: choroidal detachment (M9)  - 1/14 participants: vitreous hemorrhage/ retinal dialysis (Y3M4) |
| **Efficacy** | - 4/5 participants: BCVA improvement  - VF stable  - No significant change in macular thickness | - Significant in vision-related life quality at M3  - Vision return to base line at M12 | - 2/6 participants: BCVA improvement  - 4/6 participants: VF improvement  - Resolution of retinal hemorrhage in 1 participant with CRAO  - No changes in FFA and OCT  - ERG stable | - 2/3 participants: visual improvement  - No changes in central macular and choroidal thickness  - Severe RPE atrophy and loss of choriocapillaris | - Significant improvement in BCVA and returned to base line at M12  - 3/11 participants: central subfield thickness (CST) improvement  - 7/11 participants: CST stable  - VF stable  - ICG stable |

^a^ One study participant was excluded from the analysis at the last visit (Month 12)

AE: adverse event, AMD: age-related macular degeneration, BCVA: best corrected visual acuity, BM-MSCs: Bone marrow-derived mesenchymal stem cells, CME: cystoid macular edema, CRAO: central retinal artery occlusion, CRVO: central retinal vein occlusion, CST: central subfield thickness, ERG: electroretinogram, FFA: fundus fluorescein angiography, ICG: indocyanine green angiography, OCT: optical coherence tomography, RP: retinitis pigmentosa, RPE: retinal pigment epithelium, VF: visual field

D: day, M: month, Y: year, and N/A: not applicable
